# In Vitro Activities of Ceftobiprole, Dalbavancin, Tedizolid and Comparators against Clinical Isolates of Methicillin-Resistant *Staphylococcus aureus* Associated with Skin and Soft Tissue Infections

**DOI:** 10.3390/antibiotics12050900

**Published:** 2023-05-12

**Authors:** Sofia Maraki, Viktoria Eirini Mavromanolaki, Dimitra Stafylaki, Evangelia Iliaki-Giannakoudaki, George Hamilos

**Affiliations:** 1 Department of Clinical Microbiology and Microbial Pathogenesis, University Hospital of Heraklion, PC 71110 Heraklion, Crete, Greece; 2 School of Medicine, University of Crete, PC 71003 Heraklion, Crete, Greece

**Keywords:** skin and soft tissue infections, methicillin-resistant *S. aureus*, ceftobiprole, dalbavancin, tedizolid

## Abstract

Skin and soft tissue infections (SSTIs) are associated with significant morbidity and healthcare costs, especially when caused by methicillin-resistant *Staphylococcus aureus* (MRSA). Vancomycin is a preferred antimicrobial therapy for the management of complicated SSTIs (cSSTIs) caused by MRSA, with linezolid and daptomycin regarded as alternative therapeutic options. Due to the increased rates of antimicrobial resistance in MRSA, several new antibiotics with activity against MRSA have been recently introduced in clinical practice, including ceftobiprole, dalbavancin, and tedizolid. We evaluated the in vitro activities of the aforementioned antibiotics against 124 clinical isolates of MRSA obtained from consecutive patients with SSTIs during the study period (2020–2022). Minimum inhibitory concentrations (MICs) for vancomycin, daptomycin, ceftobiprole, dalbavancin, linezolid and tedizolid were evaluated by the MIC Test Strip using Liofilchem strips. We found that when compared to the in vitro activity of vancomycin (MIC_90_ = 2 μg/mL), dalbavancin possessed the lowest MIC_90_ (MIC_90_ = 0.094 μg/mL), followed by tedizolid (MIC_90_ = 0.38 μg/mL), linezolid, ceftobiprole, and daptomycin (MIC_90_ = 1 μg/mL). Dalbavancin demonstrated significantly lower MIC_50_ and MIC_90_ values compared to vancomycin (0.064 vs. 1 and 0.094 vs. 2, respectively). Tedizolid exhibited an almost threefold greater level of in vitro activity than linezolid, and also had superior in vitro activity compared to ceftobiprole, daptomycin and vancomycin. Multidrug-resistant (MDR) phenotypes were detected among 71.8% of the isolates. In conclusion, ceftobiprole, dalbavancin and tedizolid exhibited potent activity against MRSA and are promising antimicrobials in the management of SSTIs caused by MRSA.

## 1. Introduction

Skin and soft tissue infections (SSTIs) are among the most frequently encountered infections and have a wide range of clinical manifestations, from mild cases of erysipelas or cellulitis to life-threatening necrotizing soft tissue infections [1,2,3]. In 2013, the Food and Drug Administration (FDA) introduced a new definition for SSTIs termed “acute bacterial skin and skin structure infections” (ABSSSIs). These infections include cellulitis, erysipelas, major skin abscesses and wound infections with a minimum lesion surface area of 75 cm^2^ and accompanied by the tetrad of erythema, tenderness, edema, and warmth as local signs of infection [4]. SSTIs account for most cases of hospital admission among patients with infectious diseases, and are associated with substantial morbidity and healthcare costs [1,2,3]. In Europe, SSTIs represent 17.3% of all healthcare-associated infections [5]. In the UK and USA, complicated SSTIs (cSSTIs) account for up to 10% of all the admissions to infection units [6,7]. In the University Hospital of Heraklion, the most frequently encountered infections in surgical departments are surgical site infections [8].

*Staphylococcus aureus* is the predominant pathogen in culture-confirmed SSTIs and SSIs in hospitals and long-term care facilities (LTCFs) worldwide [9,10]. MRSA has recently become a major pathogen in patients with SSTIs. In a study of 422 patients with SSTIs presenting at emergency rooms across the USA, 59% (range 20–74%) of the cases were due to community-acquired (CA)-MRSA [11]. Similarly, among 3078 clinical isolates associated with cSSTIs from 19 countries in Europe and the Middle East, *S. aureus* was found in one third of cases and more than 50% were MRSA [12]. The increasing incidence of MRSA strains in SSTIs and the emergence of strains with multidrug resistance, including a reduced susceptibility to glycopeptides and/or linezolid, represents a global concern. The increasing rates of antibiotic resistance, especially concerning comorbidities and the risk factors for infection with MDR pathogens, makes the appropriate antibiotic selection for physicians challenging. In particular, approximately 20–25% of patients with SSTIs receive inappropriate empirical antibiotic therapy, increasing the duration of hospital stay (additional 1.39–5.4 days), the risk of hospital-acquired infections, and the patient outcome [13,14].

Treatment failure and infection relapse are both consequences of inadequate therapy in patients with CA-MRSA SSTIs. Treatment failure that necessitates modifications to the antimicrobial therapy has been more commonly encountered in nosocomial infections, in patients with co-morbidities and in complicated SSTIs [15,16]. The recurrence of the infection may occur in up to 75% of inappropriately managed SSTIs caused by CA-MRSA [17,18]. In order to improve the management of infections caused by MRSA, new antibiotics, including ceftobiprole, dalbavancin and tedizolid, have been introduced into the therapeutic armamentarium for SSTIs [19,20,21,22,23,24,25,26] (Table 1). 

The selection of appropriate empirical antimicrobial therapy should be based on the local epidemiology and susceptibility profile of MRSA to different antimicrobial agents. 

The aim of the present study is to gain insight into the in vitro activities of ceftobiprole, dalbavancin, tedizolid and comparators against recent clinical MRSA isolates associated with SSTIs.

## 2. Results

A total of 124 clinical isolates of MRSA obtained from consecutive patients diagnosed with SSTIs in our hospital during the study period were evaluated. The majority of patients were male (52.4%), and their mean age was 48.77 years (range 3–95). Of the 124 patients, approximately one-third were treated as outpatients (39.5%), 35 (28.2%) were hospitalized in surgical and pediatric surgical departments, 26 (21%) were in internal medicine departments, and 14 (11.3%) were in the pediatric department.

Among the isolates tested, high rates of resistance were evidenced for fusidic acid (64.5%), clindamycin (41.9%), levofloxacin (33.9%), and tetracycline (30.6%). The antimicrobial resistance of MRSA isolates was less common for mupirocin (16.1%) and gentamicin (6.5%); MRSA resistance was rare (1.6%) for trimethoprim-sulfamethoxazole, daptomycin and ceftaroline. Resistance to erythromycin was detected in 52 isolates (41.9%). Among them, macrolide resistance (M), constitutive clindamycin resistance (cMLS_B_) and inducible clindamycin resistance (iMLS_B_) phenotypes were found in 3.9%, 61.5% and 34.6% of isolates, respectively. The activity and susceptibility of vancomycin, daptomycin, ceftobiprole, linezolid, tedizolid and dalbavancin against the MRSA isolates from patients with SSTIs are shown in Table 2. 

All isolates were uniformly susceptible to vancomycin, teicoplanin, tigecycline, rifampicin, linezolid, tedizolid, dalbavancin and ceftobiprole. The MIC distributions of the antimicrobials tested are shown in Table 3.

The drug with the lowest MIC_90_ was dalbavancin (MIC_90_ = 0.094 μg/mL), followed by tedizolid (MIC_90_ = 0.38 μg/mL), linezolid, ceftobiprole, daptomycin (MIC_90_= 1 μg/mL), and vancomycin (MIC_90_ = 2 μg/mL). Dalbavancin demonstrated significantly lower MIC_50_ and MIC_90_ values compared to vancomycin (0.064 vs. 1 and 0.094 vs. 2, respectively, *p* < 0.001).

Tedizolid exhibited an almost threefold greater in vitro activity than linezolid. In comparison to the MIC_50_ and MIC_90_ values of tedizolid and linezolid, tedizolid had significantly lower MIC values (0.25 vs. 0.38 and 0.38 vs. 1, respectively, *p* < 0.001).

Multidrug-resistant (MDR) phenotypes were detected among 71.8% of the isolates. Table 4 shows the resistance phenotypes of the isolates tested over the entire 3-year study period. 

The predominant pattern of multidrug resistance was non-susceptibility to penicillin, oxacillin, erythromycin, clindamycin, and levofloxacin (13.5%). The second most frequent MDR phenotypes exhibited non-susceptibility to penicillin, oxacillin, fusidic acid and mupirocin (11.2%), while the third group exhibited non-susceptibility to penicillin, oxacillin, erythromycin, clindamycin, and tetracycline (10.1%).

## 3. Discussion

MRSA has become an increasingly common cause of community-acquired and healthcare-associated SSTIs. A global survey of community-acquired skin and skin-structure infections (CA-SSIs) identified MRSA as the main pathogen in 18.5% of cases, with its prevalence ranging from 15.8% in Eastern Europe to 21.4% in the Asia–Pacific region [27]. The prevalence of MRSA-related SSTIs is more than 60% in some regions of South America, Asia and the United States [28,29,30]. Ray and colleagues reported an alarming increase in the percentage of SSTIs due to MRSA, from 13% in 1998 to 48% in 2009 [31]. Similarly, Szumovski et al. demonstrated a significant increase in MRSA SSTIs between 1998 and 2005 (*p* < 0.001) [32]. The shift in the epidemiology of SSTIs is attributed to the rapid emergence of CA-MRSA strains since the late 1990s. Zervos et al. analyzed 1096 hospitalized patients with cSSTIs and reported that *S. aureus* was the prevailing pathogen in 66.4% of culture-evaluated cases, of which the most common isolate was MRSA (74.8%) [33]. A multicenter European study reported a prevalence rate of 15.1% in MRSA SSTIs, with a geographic distribution ranging from 0% in Northern European Countries to 29% in South Europe [34]. In a Greek retrospective study of 2069 SSTIs caused by *S. aureus*, MRSA represented 21% of the isolates [35]. A recent Greek multicenter survey of 1027 patients hospitalized in 16 departments with purulent cSSTIs revealed that the most common pathogen was methicillin-resistant coagulase-negative *Staphylococcus* (MRCoNS), followed by MRSA [36].

The lack of susceptibility to beta-lactams and the multidrug-resistant phenotype in MRSA makes the management of infections caused by this pathogen challenging [37]. Accordingly, MRSA is regarded as a serious threat by the Centre for Diseases Control and Prevention (CDC) and has been categorized as a high-priority multidrug-resistant (MDR) pathogen by the World Health Organization (WHO) and the Public Health Agency of Canada (PHAC) [38]. An important US study implementing whole genome sequencing identified increasing rates of MDR CA-MRSA among isolates causing SSTIs [39]. Among the isolates of the present study, more than two-thirds (71.8%) were MDR. 

While uncomplicated SSTIs respond well to oral antibiotic therapy, cSSTIs typically require intravenous antibiotic therapy. Vancomycin has been historically regarded as the gold standard regimen in the treatment of cSSTIs due to MRSA [40]; the emergence of vancomycin-resistant *S. aureus* (VRSA) and vancomycin-intermediate *S. aureus* (VISA) isolates challenges the use of vancomycin as an empirical therapy for these infections [41]. Some reports have also indicated an increasing number of MRSA isolates with high glycopeptide MICs within the susceptible range, often designated at “Vancomycin MIC Creep” [42]. This phenomenon of the “tolerance” of certain MRSA isolates to vancomycin may also account for suboptimal clinical outcomes, including reported treatment failures [41,42]. In a meta-analysis of 20 studies on MRSA SSTIs, an increased risk of treatment failure and mortality was observed in the group of patients with high MIC values for vancomycin compared with the group of patients with low MIC values [RR 1.40, 95% CI 1.15 to 1.71, and RR 1.42, 95% CI 1.08 to 1.87, respectively] [43]. Continued efforts to develop safe and effective alternatives to vancomycin have led to the development of new antimicrobials that are active against MRSA and other Gram-positive pathogens, including ceftobiprole, dalbavancin and tedizolid. 

The literature on the activity of newer antimicrobials against clinical isolates of MRSA is limited in Greece. In this study, we report on the in vitro activity of ceftobiprole, dalbavancin, and tedizolid against recent MRSA clinical isolates collected from patients with SSTIs in Greece.

Ceftobiprole is the active parent drug of the prodrug ceftobiprole medocaril, a fifth-generation cephalosporin that inhibits peptidoglycan transpeptidases by binding to penicillin-binding proteins (PBPs), including PBP2a of MRSA, and blocking the bacterial cell wall synthesis [23]. In the present study, ceftobiprole inhibited all isolates with MICs ranging from 0.064 to 1.5 μg/mL, and the concentration of ceftobiprole inhibiting 90% (MIC_90_) of the isolates was 1 μg/mL. Our results compare favorably with a US study in which 99.4% of 1643 MRSA from SSTIs were susceptible to ceftobiprole [44]. Similar high susceptibility rates (99.3%) of ceftobiprole against recent European MRSA isolates were recently reported [45]. Likewise, the ceftobiprole in vitro potency has been demonstrated in several previous studies of globally sourced MRSA isolates [46,47]. Moreover, the clinical efficacy of ceftobiprole was shown to be comparable to that of vancomycin and vancomycin plus ceftazidime in the treatment of cSSTIs in two earlier phase III clinical trials (STRAUSS I and STRAUSS II) [48]. Additionally, a randomized double-blind multicenter trial (TARGET) comparing ceftobiprole monotherapy with vancomycin plus aztreonam demonstrated that ceftobiprole is noninferior to vancomycin plus aztreonam in the treatment of ABSSSIs, in terms of early clinical response [49]. In the study of Overcash et al., the microbiological response rates were generally similar between ceftobiprole and its comparators in patients with ABSSSIs caused by MRSA [49]. In addition, ceftobiprole shares a similar safety profile to its comparators [48,49].

Dalbavancin is a semi-synthetic lipoglycopeptide antibiotic that has excellent bactericidal activity against Gram-positive pathogens, including MRSA. Unlike other glycopeptides, it has a lipophilic side chain that binds to the bacterial cellular membrane, thus enhancing its activity; it has four to eight times the potency of vancomycin. It has a longer half-life and a once-weekly dosing interval for use in the outpatient setting [20,50]. The findings of the present study demonstrated that dalbavancin showed higher activity compared with vancomycin against the MRSA isolates. Similarly, a recent systematic review reported that the overall antibacterial activity of dalbavancin on 28,539 MRSA isolates was 0.060 and 0.120 μg/mL for MIC_50_ and MIC_90_, respectively. Out of 11 studies, the pooled prevalence of dalbavancin susceptibility was 100% (95% CI: 100–100) [21]. It has been also shown that the dalbavancin MICs of VISA and heteroresistant VISA (hVISA) strains were 4- to 8-fold lower than vancomycin [51]. Moreover, dalbavancin showed a potent activity against established MRSA biofilms at concentrations achievable in the human serum and was superior to vancomycin, representing a promising therapeutic option for treating biofilm-associated SSTIs [52,53]. Additionally, the identically designed, randomized, double-blind phase III trials (DISCOVER I and DISCOVER II) demonstrated that dalbavancin had a comparable efficacy to vancomycin and linezolid in the management of ABSSSIs [54]. Of interest, dalbavancin possesses immunomodulatory properties that enhance pathogen clearance by neutrophils [55]. A multicenter, observational, retrospective, cohort study conducted in 16 hospitals, in Italy and Greece, our hospital included, found that patients with ABSSSIs receiving dalbavancin had a reduced length of hospital stay compared with those receiving other similar class intravenous antibiotics [56].

Tedizolid is a new-generation oxazolidinone with potent in vitro activity against a wide spectrum of Gram-positive bacteria, including MRSA. It exerts its antibacterial activity by binding to the 23S rRNA of the 50S subunit of the ribosome, resulting in the inhibition of protein synthesis [24]. Tedizolid differs from other oxazolidinones by possessing a modified side chain at the C-5 position of the oxazolidinone nucleus, which confers activity against certain linezolid-resistant isolates whose linezolid resistance is mediated by the *cfr* methyltransferase gene [24]. The concentration of tedizolid that inhibited 90% of the present MRSA isolates (MIC_90_) was 0.38 μg/mL, 2.6-fold lower than linezolid (MIC_90_, 1 μg/mL). Similarly, the findings of a recently published systematic review and meta-analysis demonstrated that the in vitro activity of tedizolid in 12,204 MRSA isolates was 0.25 and 0.50 μg/mL for MIC_50_ and MIC_90,_ respectively [21]. A Korean multicenter study comparing the activities of tedizolid to those of linezolid for MRSA recovered from patients with SSTIs reported that the MIC_90_ of tedizolid was 0.5 µg/mL, 4-fold lower than linezolid (MIC_90_, 2 μg/mL) [57]. The higher potency of tedizolid compared to linezolid is attributed to the ability of tedizolid, in contrast to that of linezolid, to bind to additional target site interactions within the 23S rRNA [24]. In addition, tedizolid’s efficacy advantage over linezolid against hVISA, VISA, VRSA, daptomycin-resistant *S. aureus*, and MDR phenotypes of *S. aureus* has also been shown by several investigators [24,58]. The results of the present and the aforementioned studies, along with those of clinical investigations that showed that the use of tedizolid had a more favorable profile, efficacy, and safety compared with linezolid, might warrant its use as an appropriate treatment option for MRSA infections [59,60]. Our study also demonstrated that tedizolid’s MIC_90_ (0.38 μg/mL) was lower compared to those of daptomycin and vancomycin (1 μg/mL and 2 μg/mL, respectively). Compared to vancomycin, previous studies also indicated that tedizolid’s MIC values are much lower than the MIC values of vancomycin against MRSA strains [61,62].

The present study has certain limitations, including the lack of a genetic characterization of the clinical isolates. 

## 4. Material and Methods

### 4.1. Study Design, Setting and Patient Population

All clinical MRSA strain isolates were prospectively collected from patients with skin and soft tissue infections (SSTIs), and processed by the microbiological laboratory in the University Hospital of Heraklion, Crete, Greece, from January 2020 to December 2022. The University Hospital of Heraklion is a 710-bed, tertiary care, academic hospital serving a population of 700,000 people. One isolate per patient was identified and tested. 

This study was approved by the Ethical Committee of the University Hospital of Heraklion and met the guidelines of the Helsinki declaration.

### 4.2. Bacterial Isolates

During the study period, 124 MRSA isolates collected consecutively from patients with SSTIs were studied. Only the first isolate per patient was considered. SSTIs included cellulitis, erysipelas, impetigo, folliculitis, furuncles, abscesses and carbuncles. The swabs, needle aspirates and tissue biopsies taken from the patients were promptly transported to the laboratory for microbiological analyses, including Gram stain and culture. 

Specimens were cultured onto Columbia blood, chocolate, Drigalski, Achaedler and Sabouraud dextrose agar, and incubated at 36 °C (BioMérieux, Marcy L‘Etoile, France). Isolates were identified on the basis of colony morphology, Gram stain, catalase test, coagulase test and the use of the matrix-assisted laser desorption time of flight mass spectrometry (MALDI-TOF MS) (Version 3.2) (BioMerieux).

### 4.3. Antimicrobial Susceptibility Testing

Susceptibility to penicillin, oxacillin, ceftaroline, erythromycin, clindamycin, tetracycline, tigecycline, linezolid, daptomycin, teicoplanin, vancomycin, fusidic acid, mupirocin, gentamicin, levofloxacin and trimethoprim-sulfamethoxazole was determined using the Vitek2 AST-P659 cards. Additionally, the minimum inhibitory concentrations (MICs) for vancomycin, daptomycin, ceftobiprole, dalbavancin, linezolid and tedizolid were evaluated using the MIC Test Strip and Liofilchem strips (Liofilchem, srl, Roseto degli Abruzzi, Italy), according to the manufacturer’s instructions. Briefly, Mueller–Hinton agar plates were inoculated with a 0.5 McFarland’s standard suspension of the isolate and strips were placed onto the inoculated agar plate. After incubation for 18 h at 36 °C, the MIC values were read at the intersection of the lower part of the ellipse-shaped growth inhibition area with the test strip. The breakpoints proposed by the European Union Committee on Antimicrobial Susceptibility testing (EUCAST 2022 v. 12) were used to interpret the MIC results [63]. The concurrent quality control of test procedures was performed by testing the reference strain *S. aureus* ATCC 29213.

Vancomycin, linezolid and daptomycin were chosen as comparators because these agents are commonly used for the treatment of SSTIs, especially cSSTIs and those caused by MRSA.

Isolates were phenotypically classified as methicillin-susceptible *S. aureus* (MSSA) or MRSA based on the cefoxitin disk diffusion test and the latex agglutination test for PBP2a (BioMérieux). The isolates that were resistant to erythromycin were tested for inducible clindamycin resistance via the disk approximation test (*D*-test), as per the CLSI’s recommendation [64]. In this test, a 0.5 McFarland’s standard suspension of *S. aureus* was prepared and plated onto a Mueller–Hinton agar (BioMérieux, France) plate. An erythromycin disk (15 μg) and clindamycin (2 μg) were placed 15 mm apart, edge-to-edge, on the MHA plate. Plates were analyzed after 18 h of incubation at 35 °C. The flattening of the zone of inhibition around the clindamycin, producing a “*D*” shaped blunting towards the erythromycin disk, indicated inducible resistance, and the organism was interpreted as clindamycin resistant.

MDR bacteria were defined as isolates that were non-susceptible to at least one agent in ≥3 antimicrobial categories [65].

### 4.4. Statistics

Differences between the MIC_50_ or MIC_90_ of dalbavancin and vancomycin, as well as the differences between the MIC_50_ or MIC_90_ of tedizolid and linezolid, were analyzed by a Wilcoxon matched-pairs signed rank test. Statistical significance was defined as *p* < 0.05.

## 5. Conclusions

Increasing multidrug resistance among MRSA has become a major concern regarding the treatment of cSSTIs. Our results demonstrated that ceftobiprole, dalbavancin and tedizolid are promising antimicrobial agents that complement the armamentarium to fight SSTIs by MRSA. Concomitant with the intended use of these agents, the continued surveillance of their activity is warranted in order to monitor the emergence of resistance during their use. 

## Figures and Tables

**Table 1:** Overview of novel antibiotics with activity against MRSA approved by FDA and/or EMA.

Antibiotic	Antibiotic Class	Mechanism of Action	Type of Activity	Antimicrobial Spectrum	Date of Approval	Reference
Dalbavancin	Lipoglycopeptide	Inhibits bacterial cell wall synthesis by binding to D-alanyl-D-alanyl residue on growing peptidoglycan	Bactericidal	MSSA, MRSA, VISA, *S. pyogenes*, *S. agalactiae*, *S. anginosus*, *S. faecalis* vancomycin-susceptible	FDA: May 2014	[19,20,21]
Telavancin	Lipoglycopeptide	Inhibits peptidoglycan cell wall synthesis and disrupts bacterial cell membrane integrity	Bactericidal	MSSA, MRSA, hVISA, VISA, *S. pyogenes*, *S. agalactiae*, *S. anginosus* group, PRSP, VSE	FDA: September 2009	[20]
Oritavancin	Lipoglycopeptide	Inhibits peptidoglycan cell wall synthesis and disrupts bacterial cell membrane integrity	Bactericidal	MSSA, MRSA, VISA, VRSA, VRE	FDA: August 2014 EMA: March 2015	[20]
Ceftaroline	Fifth–generation cephalosporin	Inhibits cell wall synthesis by binding to penicillin-binding proteins (PBPs)	Bactericidal	MSSA, MRSA, VRSA, *S. pyo-genes*, *S. agalactiae*, *S. pneumoniae*, *E. faecalis*, Gram-negative bacteria (except *Pseudomonas* and ESBL Enterobacterales)	FDA: October 2010 EMA: August 2012	[22]
Cefobiprole	Fifth–generation cephalosporin	Inhibits cell wall synthesis by binding to penicillin-binding proteins (PBPs)	Bactericidal	MSSA, MRSA, ampicillin-susceptible enterococci, PRSP, Gram-negative bacteria (except pathogens producing ESBLs)	EMA: October 2013	[23]
Tedizolid	Second- generation oxazolidinone	Inhibits bacterial protein synthesis by binding to the 50 S ribosomal subunit	Bacteriostatic	MSSA, MRSA, CoNS, *S. pyogenes*, *S. agalactiae*, *S. anginosus* group, VSE, VRE	FDA: June 2014 EMA: March 2015	[24]
Omadacycline	New-generation Broad-spectrum aminomethylcycline	Inhibits bacterial protein synthesis by binding to the 30 S ribosomal subunit	Bacteriostatic	MSSA, MRSA, PRSP, VSE, VRE	FDA: October 2018	[25]
Delafloxacin	New-generation anionic fluoroquinolone	Inhibits the activities of both bacterial topoisomerase IV and DNA gyrase	Bactericidal	MRSA, MSSA, CoNS, *S. pyogenes*, *S. agalactiae*, *S. anginosus* group, *S. pneumoniae*, *E. coli*, *K. pneumoniae*, *E. cloacae*, *P. aeruginosa*	FDA: June 2017	[26]

FDA, US Food and Drug Administration; EMA, European Medicines Agency; MSSA, methicillin-susceptible *S. aureus*; MRSA, methicillin-resistant *S. aureus*; hVISA, heteroresistant vancomycin-intermediate *S. aureus*; VISA, vancomycin-intermediate *S. aureus;* VRSA, vancomycin-resistant *S. aureus*; VSE, vancomycin-susceptible enterococci; VRE, vancomycin-resistant enterococci; PRSP, penicillin-resistant *S. pneumoniae*; CoNS, coagulase-negative staphylococci; ESBL, extended-spectrum beta-lactamase.

**Table 2:** Activity of antimicrobial agents against 124 MRSA isolates collected from patients with SSTIs in Greece (2020–2022).

Antibiotic	MIC_50_	MIC_90_	Range	S%
Vancomycin	1	2	0.38–2	100
Daptomycin	0.5	1	0.125–1.5	98.4
Ceftobiprole	0.38	1	0.064–1.5	100
Linezolid	0.38	1	0.125–2	100
Tedizolid	0.25	0.38	0.094–0.5	100
Dalbavancin	0.064	0.094	0.008–0.125	100

**Table 3:** Distribution of minimum inhibitory concentration values of the antimicrobial agents tested against the MRSA isolates from SSTIs.

Number of Isolates Inhibited at (mg/L)															
Antibiotic	0.008	0.023	0.032	0.047	0.064	0.094	0.125	0.19	0.25	0.38	0.5	0.75	1	1.5	2
Vancomycin										2	5	16	43	36	22
Daptomycin							2	4	24	12	20	12	48	2	
Ceftobiprole					4		5	12	19	28	28	12	4	12	
Linezolid							10	10	36	20	8	4	30	2	4
Tedizolid						4	28	28	30	22	12				
Dalbavancin	2	14	8	30	40	20	10								

**Table 4:** Resistance phenotypes of the *S. aureus* isolates to antimicrobials over the 3-year period.

		No.
	**Resistance to three indicated classes**	**26**
1	P-OX, FA, MU	10
2	P-OX, E, CM	6
3	P-OX, FA, LE	4
4	P-OX, GM, FA	2
5	P-OX, E, MU	2
6	P-OX, FA, TE	2
	**Resistance to four indicated classes**	**35**
7	P-OX, E, CM, LE	12
8	P-OX, E, CM, TE	9
9	P-OX, FA, GM, TE	8
10	P-OX, CM, FA, MU	2
11	P-OX, FA, MU, LE	2
12	P-OX, E, FA, LE	2
	**Resistance to five indicated classes**	**16**
13	P-OX, E, CM, FA, LE	8
14	P-OX, E, CM, FA, TE	6
15	P-OX, CM, TE, LE, SXT	2
	**Resistance to six indicated classes**	**12**
16	P-OX, E, CM, FA, LE, TE	8
17	P-OX, E, CM, FA, LE, MU	4

P-OX, penicillin-oxacillin; E, erythromycin; CM, clindamycin; FA, fusidic acid; MU, mupirocin; LE, levofloxacin; TE, tetracycline; GM, gentamicin; SXT, trimethoprim-sulfamethoxazole.

## Data Availability

The data presented in this study are available on request from the corresponding author.
